# The elevated admission white blood cell count relates to adverse surgical outcome of acute Stanford type a aortic dissection

**DOI:** 10.1186/s13019-020-1078-5

**Published:** 2020-03-14

**Authors:** Mingjia Ma, Juan Shi, Xin Feng, Jing Wang, Ligang Liu, Xiang Wei

**Affiliations:** Division of Cardiothoracic and Vascular Surgery, Tongji Hospital, Tongji Medical College, Huazhong University of Science and Technology, 1095# Jiefang Ave. Wuhan, Wuhan,, 430030 China

**Keywords:** White blood cell count, Acute Stanford type a aortic dissection, Surgical outcome, Circulatory arrest, Risk factors

## Abstract

**Background:**

The purpose of the study was to examine the association between white blood cell count (WBCc) on admission and early outcome in patients with the acute Stanford type A aortic dissection (TAAD).

**Methods:**

From January 2012 to December 2018, we retrospectively evaluated a series of 331 consecutive patients underwent surgery for TAAD in Tongji Hospital. The patients were divided into 2 groups based on the WBCc, i.e. the normal WBCc group (WBCc≤11 × 10^9^/L) and leukocytosis group (WBCc> 11 × 10^9^/L). The perioperative data were compared between the 2 groups. The in-hospital mortality and the compositive adverse event including multi-organ dysfunction syndrome, postoperative stroke, tracheotomy, and re-exploration for stopping bleeding were set as end points. Cox regression were used to assess the potential risk factors.

**Results:**

The in-hospital mortality was nearly 3 time higher in the leukocytosis group than in the normal WBCc group (20.9% vs.8.1%, *P* = 0.001), and 15.1% overall. For the circulatory arrest, there was significant higher mortality in patients with leukocytosis than normal WBCc group (26.1%vs.8.9%, *P* = 0.001). After adjustment for confounding factors, the leukocytosis was found to be a strong independent predictor of in-hospital mortality (odds ratio = 3.10; 95% confidence interval 1.38 to 6.97, *P* = 0.006). The leukocytosis was also a risk factor of adverse events (odds ratio = 1.80; 95% confidence interval 1.07 to 3.04, *P* = 0.027).

**Conclusions:**

The WBCc within 24 h of admission for TAAD is a strong and independent predictor of in-hospital mortality as well as short-term clinical events. The results of this study have important clinical implications for risk-stratifying patients with TAAD.

## Background

Acute Stanford type A aortic dissection (TAAD) is cardiovascular disaster associated with high mortality. The surgical management is the most effective therapy. However, despite advances in the technique and perioperative care, the surgical mortality and morbidities of potentially fatal complications of TAAD stayed relatively high, and it may be affected by the preoperative status of the patients [1]. Therefore, efforts have been put to identify high-risk TAAD patients [2, 3]. Several predictors of adverse events in acute aortic dissection have been discussed [4]. To our knowledge, the special impact of the white blood cell count (WBCc) on surgical outcome of acute aortic dissection remained unelucidated. The present study was designed to assess the relationship between WBCc and surgical outcomes in TAAD patients.

## Methods

From January 2012 to December 2018, a series of consecutive patients underwent surgery for acute Stanford type A aortic dissection in Tongji hospital. The chart review was conducted to gathering clinical information. TAAD was defined as acute if onset of the symptoms within 2 weeks before admission and the diagnosis was confirmed by computed tomographic angiography. We excluded the cases of iatrogenic aortic dissection, chronic aortic dissection, and patients who had a history of cardiac surgery. Three cases of aortic dissection combined with pregnancy were also excluded. The primary end point was in-hospital mortality and the second end point were the compositive adverse events comprised multi-organ dysfunction syndrome, postoperative stroke, tracheotomy, and re-exploration for stopping bleeding. The patient that has one or more adverse events was count as an adverse event case.

The patients were divided into normal WBCc group (WBCc≤11 × 10^9^/L) and elevated WBCc group (WBCc> 11 × 10^9^/L). The perioperative data were compared between the 2 groups. This study was approved by the local clinical research ethics committee and the consent of the subjects were waived.

### Definition

Primarily, the WBCc on admission above 11 × 10^9^/L was defined as leukocytosis [5]. Emergent surgeries were operations carried out within 24 h after admission. Acute renal dysfunction was defined as elevation of serum creatinine level 3-fold greater than baseline or a new requirement for transient hemodialysis. Acute hepatic insufficiency was defined as elevation of aspartate aminotransferase greater than 200 IU/L. Hypoxemia was defined as oxygenation impairment with arterial partial pressure of oxygen/fraction of inspired oxygen ratio (PaO_2_/FiO_2_) < 300 and acute respiratory dysfunction was PaO_2_/FiO_2_ < 150 that occurred on admission. Transient neurologic dysfunction was defined as resolvable mental disorder without identifiable brain imaging change after operation. Permanent neurologic dysfunction (PND) was neurologic deficit with detectable change in magnetic resonance imaging or computed tomography scans.

### Surgical technique

All the operation was performed via median sternotomy approach. The cardiopulmonary bypass (CPB) was established by arterial cannulation of unilateral femoral artery and/or the right subclavian artery and venous cannulation in right atrium. A left ventricular vent line was inserted through the right superior pulmonary vein.

After cross clamping the ascending aorta, the aorta was longitudinally opened and cardioplegic solution was directly administrated via the coronary ostia for myocardia protection. The lesion of the dissection and geometrical morphology of root were carefully detected. If necessary, resuspension of cusps with commissural stitches or aortic root replacement with composite prosthesis was performed depending on the lesion of the sinus and dilatation of aortic root. The coronary artery bypass graft was performed in circumstances of coronary artery ostia teared or combined severe coronary artery disease.

After the aortic root procedure was completed, the distal extent of the aortic replacement was determined by the location of the intimal tear. When the intimal tear was limited in the ascending aorta, the ascending aorta replacement was performed. If the primary tear located in the arch or the intimal tear extended into aortic arch, hemi-arch replacement or total aortic arch replacement combined with stent frozen elephant trunk implantation was performed under circulatory arrest. During circulatory arrest, the systemic hypothermia maintained at moderate (20–28 °C) level at the rectal site, and antegrade selective cerebral perfusion was applied for neuroprotection. In year 2012, arch debranching with intraoperative endograft deployment was introduced for total arch repair. The surgical method of individual case was determined by both the lesion and surgeon’s personal preference and experience.

### Statistical analysis

Continuous variables are presented as the mean ± standard deviation, while non-normally distributed variables are presented as the median and interquartile ranges. Normally distributed variables are compared using Student’s t test. The Mann-Whitney U test is used for non-normally distributed data. The categorical data are calculated by chi-square test in different groups. For chi-square contingency-table test, the results were interpreted by standardized residual method of post hoc analysis [6]. Univariable and multivariable Cox regression are used to assess the potential risk factors of in-hospital mortality and major adverse event cases. Adjusting parameters and variables those known as risk factors or variables with *P* value less than 0.1 in univariate analysis are included in the multivariate proportional hazards models. We built two kinds of multivariable Cox regression models, the WBCc is treated as the dependent variable in the form of quartiles (model 1) and two-category data (model 2). The cut-off points of WBCc are evaluated by receiver operating characteristic (ROC) curves. Statistical significance was set as a two-sided *P*-value < 0.05. SPSS Statistics 19.0 (IBM, Somers, NY) was used for statistical analysis.

## Results

There were 331 patients included in this study. The male to female ratio was 4:1. The age of the patients was 48.3 ± 9.4 years (ranged 25–72 years). Nearly half of the operations (165 cases, 49.8%) were performed emergently.

**Fig. 1:**
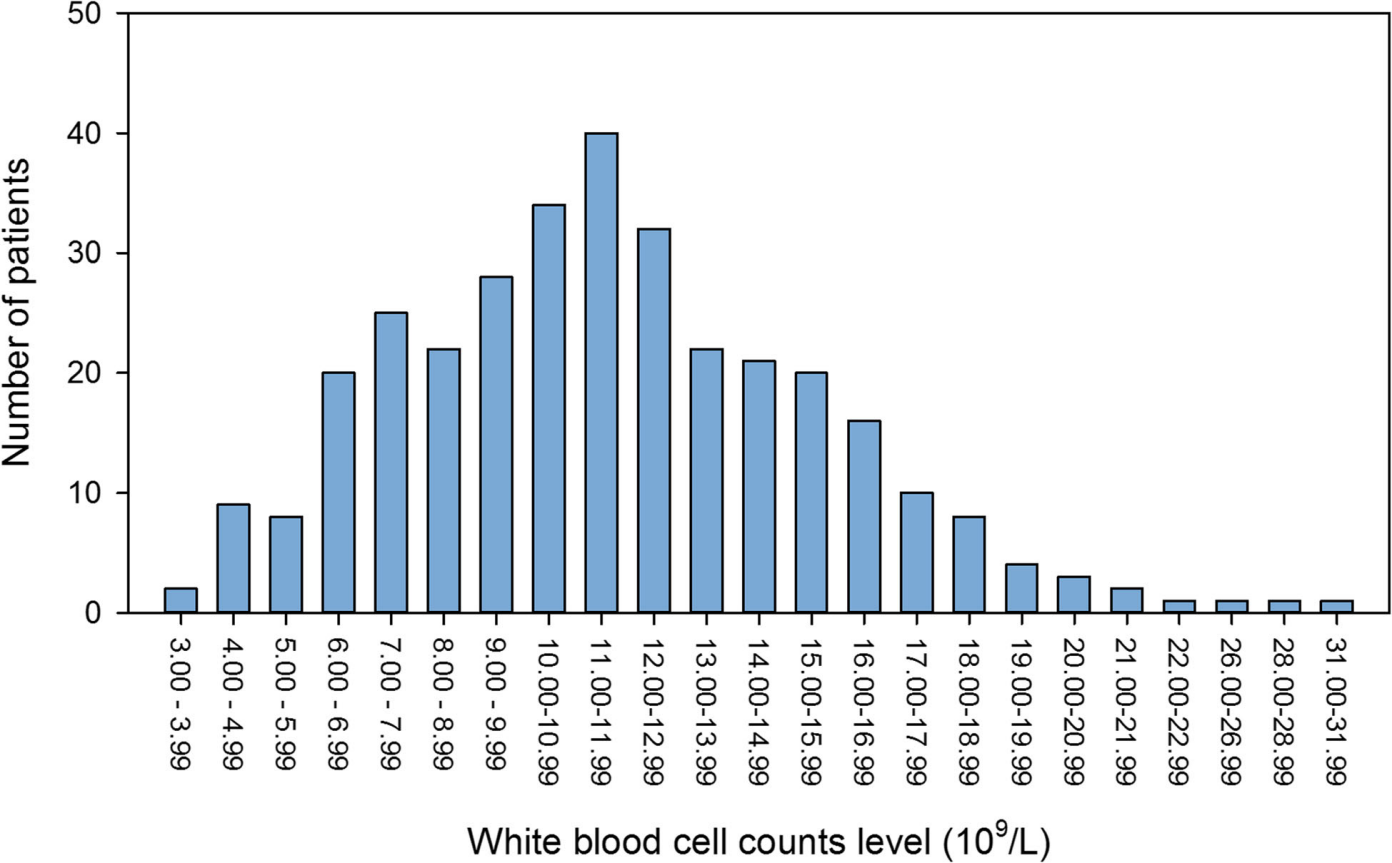
Distribution of the white blood cell count on admission

The mean WBCc was 11.69 ± 4.11 × 10^9^/L, and distribution of individual WBCc levels in every 1 × 10^9^/L were displayed in Fig. 1. Compared to the patients in the group of the normal WBCc (normal group, *n* = 149, 45%), patients in the group of the elevated WBCc (leukocytosis group, *n* = 182, 55%) were more likely to be younger (50.2 ± 9.5 years vs.46.7 ± 9.2 years, *P* = 0.001). In addition, the patients in leukocytosis group had higher neutrophil-to-lymphocyte ratio (7.0 ± 5.3 vs. 12.2 ± 6.6, *P* < 0.001) on admission and underwent emergent surgery more frequently (36.2% vs. 61.0%, P < 0.001). Interesting, the signs end-organ ischemia/insufficient involved heart, lung, and kidneys were similar observed both in the normal WBCc group and leukocytosis WBCc group, but the incidence of acute hepatic injury was found only in the leukocytosis WBCc group (*n* = 10). The incidence of hypertension was not significant different between the two WBCc groups. The preoperative data were presented in Table 1.

**Table 1:** Democratic data

Variables	Group I	Group II	*P* value
	*n* = 149	*n* = 182	
Gender Male (%)	111 (74.5)	151 (83.0)	0.059
Age (years)			
Mean ± SD	50.2 ± 9.5	46.7 ± 9.2	**0.019**
Median (IQR)	50.0 (43.0–57.0)	47.0 (41.0–51.0)	
Diabetes mellitus (%)	13 (8.7)	16 (8.8)	0.983
Hypertension (%)	110 (73.8)	139 (76.4)	0.593
Body mass index			
Mean ± SD	23.9 ± 3.9	25.7 ± 3.6	**< 0.001**
Median (IQR)	23.9 (21.0–25.7)	25.5 (23.2–27.8)	
Triglyceride (mmol/L)			
Mean ± SD	1.1 ± 0.7	1.2 ± 0.7	**0.030**
Median (IQR)	1.0 (0.7–1.4)	1.1 (0.8–1.4)	
WBCc (×10^9^/L)			
Mean ± SD	8.3 ± 1.9	14.5 ± 3.2	**< 0.001**
Median (IQR)	8.4 (6.9–9.9)	13.8 (12.1–16.0)	
Hypoxemia (%)	67 (45.0)	108 (59.3)	**0.009**
Acute respiratory dysfunction (%)	6 (4.0)	12 (6.8)	0.278
Heart ischemia (%)	35 (23.5)	43 (23.6)	0.977
Acute hepatic injury (%)	0	10 (5.5)	**0.003**
Renal disfunction (%)	6 (4.0)	12 (6.6)	0.306
Emergency (%)	54 (36.2)	111 (61.0)	**< 0.001**

*IQR* interquartile range, *SD* standard deviation, *WBCc* white blood cell count

Group I: normal WBCc group, Group II: leukocytosis group

Note: *P*-value< 0.05 is highlighted in bold type

As a whole, the mean operation time was 503.3 ± 116.5 min and the mean cardiopulmonary bypass time was 225.4 ± 61.8 min. The cross-clamping time was 120.0 ± 31.6 min. The primary intimal tears were identified by directly inspection and resected. The arch-involved procedures were performed in 298 patients, including 228 total arch replacements, 20 hemiarch replacements and 50 aortic arch debranching procedures. Concomitant coronary artery bypass grafting was performed in 25 patients. Overall, 17 patients required a re-sternotomy because of hemorrhagic problems. Ten patient developed sternal infection, of them, 7 cases (70.0%) had leukocytosis before surgery. Comparing the patients in the normal group to the leukocytosis group, more total arch procedures were performed in the leukocytosis group (61.7% vs.74.7%, *P* = 0.011). The high WBCc level seem to be related to serious aortic involvement. There was no significant difference of operation time, cardiopulmonary bypass time and Cross-clamping time between the two groups. The operation data were displayed in Table 2. We then divided patients into 4 groups based on the quartiles of their WBCc on admission (i.e. quartile 1: < 8.79 × 10^9^/L; quartile 2: 8.80–11.49 × 10^9^/L; quartile 3: 11.5–14.49 × 10^9^/L; and quartile 4: > 14.50 × 10^9^/L). When we compared the patients among the 4 quartiles groups, more cases of intimal tear extended into the aortic root were observed in the highest quartiles group than the other 3 quartiles groups (Table 3).

**Table 2:** Operation data

Variables	Group I	Group II	*P* value
	*n* = 149	*n* = 182	
Sinus tear (%)	33 (22.1)	55 (30.2)	0.098
Bentall (%)	46 (30.9)	61 (33.5)	0.609
CABG (%)	12 (8.1)	13 (7.1)	0.755
Operation time (min)			
Mean ± SD	494.4 ± 110.0	510.7 ± 121.4	0.837
Median (IQR)	494.0 (421.5–560.0)	501.0 (435.0–572.0)	
CPB time (min)			
Mean ± SD	221.8 ± 60.4	228.3 ± 62.9	0.193
Median (IQR)	217.0 (184.5–251.0)	223.5 (200.0–254.3)	
Cross-clamping (min)	120.2 ± 33.0	119.9 ± 30.6	0.937
AVR (%)	86 (57.7)	114 (62.6)	0.363
HAR (%)	13 (8.7)	7 (3.8)	0.064
TAR (%)	92 (61.7)	136 (74.7)	**0.011**
Arch debranching (%)	26 (17.4)	24 (13.2)	0.281
Transfusion			
RBC (U)			
Mean ± SD	7.0 ± 6.4	8.4 ± 7.5	0.549
Median (IQR)	5.3 (3.0–9.6)	7.0 (3.5–11.0)	
Plasma (ml)			
Mean ± SD	946.0 ± 761.6	1278.6 ± 1110.8	**0.036**
Median (IQR)	900.0 (300.0–1400.0)	900.0 (450.0–1800.0)	

*AVR* aortic valve resuspension, *CABG* coronary artery bypass graft, *CPB* cardiopulmonary bypass, *HAR* hemi-arch replacement, *IQR* interquartile range, *SD* standard deviation, *TAR* total arch replacement, *RBC* red blood cell

Group I: normal WBCc group, Group II: leukocytosis group

Note: *P*-value< 0.05 is highlighted in bold type

**Table 3:** Comparison of operation data among 4 quartiles of white blood cell count groups

Variables	Q1	Q2	Q3	Q4	*P* value*	
	*n* = 83	*n* = 85	*n* = 87	*n* = 76	overall	(Q1, Q2, Q3) vs. Q4
Sinus tear	19 (22.9)	19 (22.4)	20 (23.0)	30 (39.5)	**0.038**	**0.004**
TAR	47 (56.6)	58 (68.2)	63 (72.4)	60 (78.9)	**0.019**	0.028

*Q* quartiles of white blood cell count on admission, *TAR* total arch replacement. Q1: < 8.79 × 10^9^/L; Q2: 8.80–11.49 × 10^9^/L; Q3: 11.5–14.49 × 10^9^/L; and Q4: > 14.50 × 10^9^/L

Note: *P*-value< 0.05 is highlighted in bold type.* The post hoc tests are processed with *P* value< 0.013 as statistical significance, adjusted by the Bonferroni correction

The in-hospital mortality was 15.1% overall. The mortality in the normal group was significantly lower than that in the leukocytosis group (8.1% vs. 20.9%, *P* = 0.001). Thirty-three patients had the postoperative PND, and 2/3 of them had leukocytosis preoperatively. There was a trend of increasing risk of in-hospital mortality and comorbidities of PND and multi-organ dysfunction symptoms with increasing WBCc. The duration of ventilation time and intensive care unit stay time were significantly longer in the leukocytosis group compared with patients did in the normal WBCc group, respectively (Table 4). After excluding the dead cases, we could not find a significant relationship between the preoperative WBCc and total in-hospital time.

**Table 4:** Outcomes

Variables	Group I	Group II	*P* value
	*n* = 149	*n* = 182	
In-hospital mortality (%)	12 (8.1)	38 (20.9)	**0.001**
TND (%)	42 (28.2)	65 (35.7)	0.145
PND (%)	12 (8.1)	21 (11.5)	0.292
Ventilation time (h)			
Mean ± SD	94.9 ± 107.4	137.8 ± 131.6	**< 0.001**
Median (IQR)	46.0 (18.0–139.5)	108.0 (31.3–196.0)	
Tracheotomy (%)	23 (15.4)	41 (22.5)	0.104
Re-exploration (%)	4 (2.7)	13 (7.1)	0.068
MODS (%)	7 (4.7)	18 (9.9)	0.075
RRT (%)	19 (12.8)	26 (14.3)	0.685
ICU time (h)			
Mean ± SD	139.4 ± 94.5	177.6 ± 132.9	**0.009**
Median (IQR)	115.0 (68.0–185.3)	161.5 (81.3–239.3)	
In hospital time (d)*			
Mean ± SD	29.0 ± 14.7	26.0 ± 11.7	0.062
Median (IQR)	27.0 (21.0–34.5)	24.0 (18.0–30.0)	

*ICU* time intensive care unit stay time, *IQR* interquartile range, *MODS* multi-organ dysfunction syndrome, *PND* permanent neurologic dysfunction, *RRT* renal replacement therapy, *SD* standard deviation, *TND* transient neurologic dysfunction

Group I: normal WBCc group, Group II: leukocytosis group

Note: *P*-value< 0.05 is highlighted in bold type

* The variable in hospital time was analyzed after exclusion of the in-hospital dead cases

**Fig. 2:**
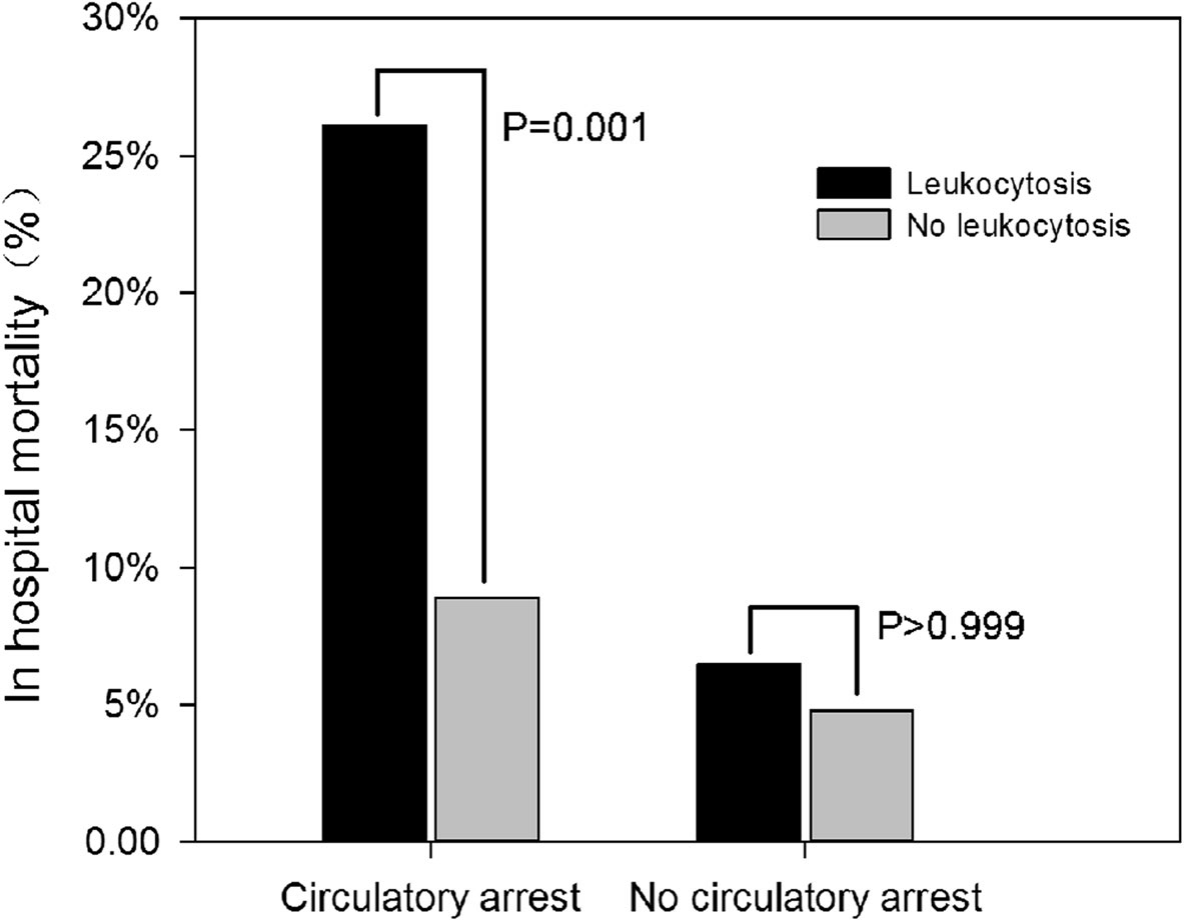
The in-hospital mortality comparisions between patients with/without leukocytosis (WBCc> 11 × 10^9^/L), *P* values were obtained by chi-square test. In the subset of patients with circulatory arrest, the difference of in-hospital mortality was significant (*P* = 0.001)

In the subgroup analysis of patients underwent surgery with /without circulatory arrest, there are significant differences for in-hospital mortality between the preoperative leukocytosis and no leukocytosis. For the circulatory arrest group, there was significant higher mortality in patients with leukocytosis than normal WBCc group (26.1% vs.8.9%, *P* = 0.001). However, this difference between leukocytosis and normal WBCc was not found (6.5% vs. 4.8%, *P* > 0.999) in the patients without circulatory arrest (Fig. 2).

In univariate analysis, the leukocytosis was risk factor of either in-hospital mortality or compositive adverse events (Table 5). By multivariable-adjusted Cox regression analysis, the preoperative WBCc levels stratified by quartiles and renal dysfunction were found as predictor of in-hospital mortality. The prolonged cardiopulmonary bypass (CPB) time was also an independent risk factor associated with in-hospital mortality when considered as a continuous variable. For the composite endpoint of major adverse events, the elevated WBCc on admission was a significant risk factor of the multivariable analysis. The predictors of 30-day mortality and adverse events according to multivariate analysis were presented in Table 6.

**Table 5:** Univariate analysis

Variables	Mortality			Adverse event		
	*P*	HR	95%CI	*P*	HR	95%CI
Gender	0.200	1.739	0.750–4.060	**0.050**	1.806	0.999–3.265
Age	0.775	0.995	0.960–1.030	0.871	0.998	0.975–1.022
BMI	**0.002**	1.138	1.049–1.234	**< 0.001**	1.127	1.059–1.200
Emergency	0.741	1.107	0.606–2.021	0.339	1.245	0.794–1.950
WBCc	**0.005**	1.105	1.030–1.185	**0.001**	1.101	1.040–1.166
CPB time	**< 0.001**	1.015	1.009–1.021	**< 0.001**	1.010	1.005–1.014
Cross-clamping time	**0.030**	1.011	1.001–1.020	**0.050**	1.007	1.000–1.014
Diabetes mellitus	0.382	1.53	0.589–3.970	0.836	0.919	0.412–2.046
Hypertension	0.622	1.199	0.583–2.466	0.470	1.214	0.717–2.058
Operation type	0.282	1.259	0.828–1.915	0.550	1.094	0.816–1.467
Renal dysfunction	**0.001**	5.162	1.928–13.821	**0.009**	3.796	1.386–10.395

*BMI* body mass index, *CI* confidence interval, *CPB* cardiopulmonary bypass, *HR* hazard ratio, *WBCc* white blood cell count. Note: *P*-value< 0.05 is highlighted in bold type

**Table 6:** Multivariate analysis

In-hospital M	HRs	95%CI	*P* value	Adverse event	HRs	95%CI	*P* value
Model 1							
Quartile 1	1.000			Quartile 1	1.000		
Quartile 2	3.524	0.847–14.665	0.083	Quartile 2	1.577	0.758–3.282	0.223
Quartile 3	8.864	2.201–35.693	**0.002**	Quartile 3	1.764	0.843–3.692	0.132
Quartile 4	7.579	1.832–31.351	**0.005**	Quartile 4	2.257	1.048–4.864	**0.038**
CPB	1.018	1.010–1.026	**< 0.001**	CPB	1.010	1.004–1.016	**0.001**
Cross-clamping	1.006	0.992–0.979	0.272	Cross-clamping	0.995	0.986–1.006	0.376
Renal dysfunction	6.955	2.126–22.756	**0.001**	Renal dysfunction	4.029	1.365–11.889	**0.012**
				BMI	1.090	1.017–1.169	**0.015**
Model 2							
Elevated WBCc	3.104	1.382–6.972	**0.006**	Elevated WBCc	1.801	1.068–3.037	**0.027**
CPB	1.018	1.010–1.026	**< 0.001**	CPB	1.011	1.005–1.017	**< 0.001**
Cross-clamping	0.993	0.980–1.007	0.333	Cross-clamping	0.995	0.985–1.005	0.371
Renal dysfunction	6.718	2.132–21.169	**0.001**	Renal dysfunction	4.011	1.373–11.719	**0.011**
				BMI	1.089	1.016–1.168	**0.016**

*BMI* body mass index, *CI* confidence interval, *CPB* cardiopulmonary bypass, *HR* hazard ratio, In-hospital *M* in-hospital mortality, *WBCc* white blood cell count. Note: *P*-value< 0.05 is highlighted in bold type

**Fig. 3:**
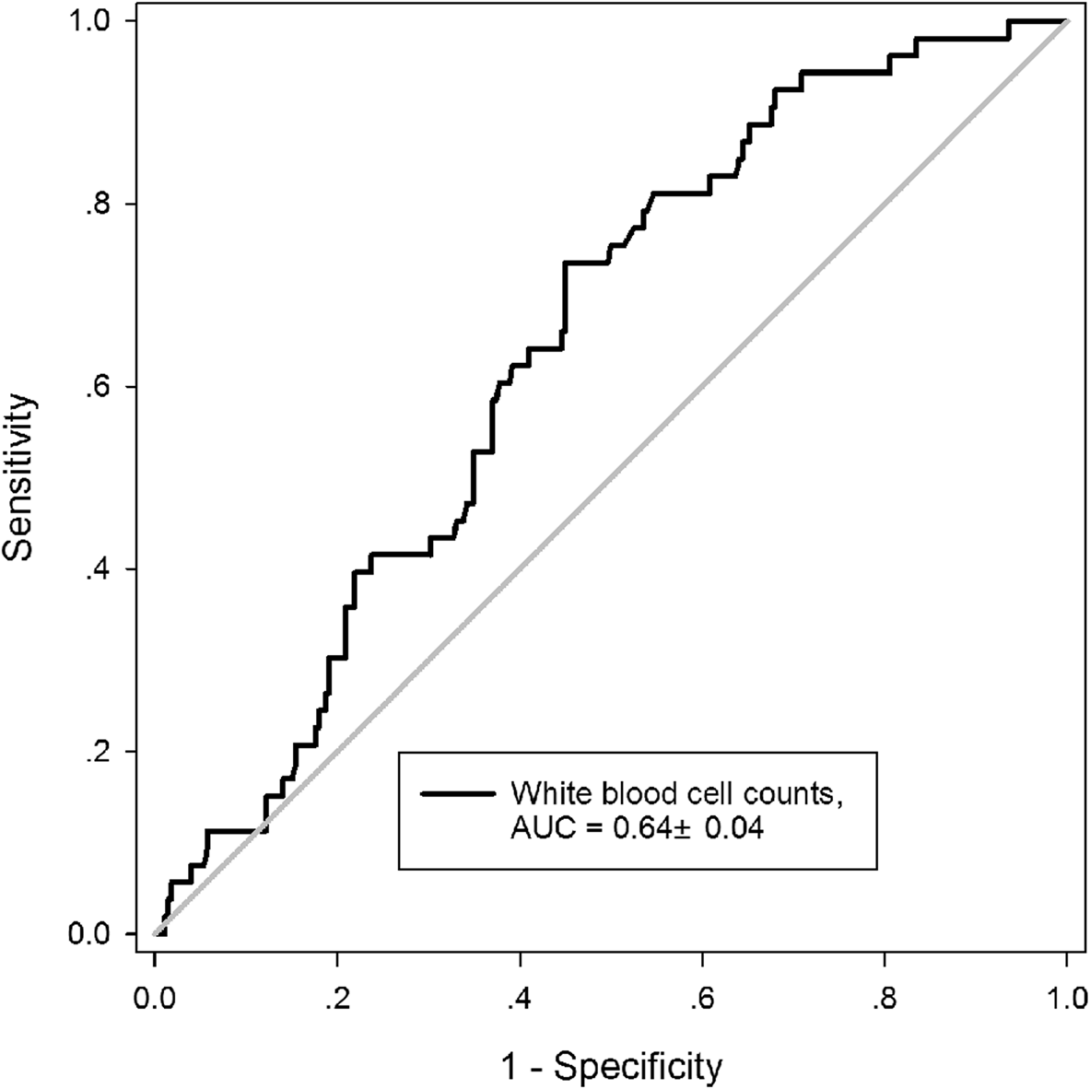
Receiver operating characteristic (ROC) curve for the prediction by admission white blood cell count of in-hospital mortality in patients underwent surgery for acute type A aortic dissection. AUC, area under ROC curve

The ROC curve analysis displayed that WBCc beard a significant relation to in-hospital mortality in TAAD patients (area under the curve 0.64 ± 0.04, *P* = 0.001, Fig. 3). The cut-off value is 11.44 × 10^9^/L with sensitivity of 73.6% and specificity of 55.0%.

## Discussion

In this study, the clinical data of a series of patients underwent surgery for acute aortic dissection were assessed. In a 7-year experience at a single institution, an in-hospital mortality of less than 16% was achieved. The high WBCc level on admission was related to high in-hospital mortality and as a risk factor of a composite adverse event involved heart, brain, lung and systemic condition. To the best of our knowledge, this is the first study focused on the common index of WBCc effect on surgical outcome of acute type A aortic dissection.

According to data from registry studies, the early mortality of large cohorts ranged 19.6–25.1% [1, 7], and our result was similar to them. Given the advances of the surgical technique and surgeons’ experiences, it may continue to remain a challenge to further improve the early outcome in TAAD patients. Due to the threatening nature and devastating presentation of the disease, the outcomes of surgical management have been supposed to be partially influenced by the patients’ preoperative status. A numerous of studies have analyzed risk factors as the primary determinants of outcomes for TAAD surgery. The reported risk factors included advanced age, evidences of complications such as neurological deficit, myocardial ischemia, shock, kidney failure, and limb ischemia et al. [1, 8–11]. In the present study, preoperative renal dysfunction, the elevated WBCc on admission, and prolonged CPB time were associated with early mortality and morbidities of composite adverse events in surgical intervention of TAAD.

Inflammatory process plays an important role in the development of aortic dissection. High inflammatory biomarker has been observed in patients as soon as the onset of the syndrome, suggesting a very early initiation of a cascade of inflammation in TAAD [12]. The aortic tissue destruction and thrombi in the false lumen created by the dissection may induce the inflammatory reaction. The activated circulating WBCs adhered to endothelium and damaged it with toxic oxygen compounds and proteolytic enzymes, this contributed a lot to the injury of the tissues. The WBC such as neutrophil and macrophages have been detected in the teared aortic tissues [13].

On the other hand, the acute aortic dissection might cause malperfusion syndrome. Significant WBCc elevation has been observed in the end-organ ischemic complications those resulted from cerebral, visceral, or coronary malperfusion [14, 15]. Higher WBCc in patients with TAAD might reflect the higher severity of malperfusion syndromes when the end-organ involved. Takahashi, T. found WBCc on admission was significant higher in acute aortic dissection patients with relatively severe acute kidney injury when compared with patients without acute kidney insufficiency [16]. The patients with high preoperative WBCc had poor prognosis. Guan et al. demonstrated that WBCc was a significant predictor to type A aortic dissection patients with postoperative neurological complications [17]. The WBCc level indicated the severity of the aortic dissection and the organ malperfusion. In this study, there was significant positive relationship between WBCc and organ malperfusion. Furthermore, since the immediate surgery has been recommended to prevent aortic rupture, improve coronary, cerebral, and visceral circulation, it is comprehensible that the emergent case was more frequently performed in the elevated WBCc group than that in the normal WBCc group in this study.

The surgical modality for TAAD has evolved in the past decades. In this study, we could not find the surgical manners as a significant risk factor for the 30-day mortality and adverse event. That means, the surgical expertise, though evolved with time, remained comparatively stable. However, in the subgroup analysis, the patients with preoperative leukocytosis underwent circulatory arrest displayed worse surgical outcomes than the patients without circulatory arrest. The preoperative high WBCc level might relate with pathophysiological change of organs during circulatory arrest. The potential explanation maybe circulatory arrest process prolonged the CPB time and aggravate inflammatory response [18]. In the animal model, the inflammation cascades were provoked during rewarming stage after circulatory arrest [19]. The inflammatory cytokines and cells might add insult to ischemia-reperfusion injury during the surgery, and lead to poor prognosis.

The elevated WBCc on administration had related to risk of respiratory complications of TAAD. Duan et al. reported a series of 172 TAAD patients, according to their experience, the inflammatory cells and cytokines such as WBCc and neutrophil counts; levels of C-reactive protein and interleukin-6 were responsible for the hypoxemia before surgery, and the remission of hypoxemia can be achieved by administration of anti-inflammatory agent Ulinastatin [20]. On the other hand, the system inflammatory response that attacked the lungs increased the risk of respiratory infection. Respiratory complications were important causes of surgical mortality for TAAD [21]. In our own data, prolonged mechanical ventilation assistance time after surgery was also associated with elevated preoperative WBCc. Considering the peripheral WBCc was a very common index for both the infections and inflammatory processes in the acute stage of aortic dissection, it might be an optimal predictor of respiratory complications. In our center, the managements to improve pulmonary function such as emergency intubation on admission have been actively applied in the high-risk patients to reduce surgical mortality and perioperative complication morbidity.

### Limitation

This is a retrospective study enrolled the patient’s clinical data in single institution. Although the blood samples of patients were collected as soon as on admission, the interval between symptom onset and the first blood routine examination could not be accurately evaluated. We included a relatively small number of patients who underwent surgeries in a single center, it might create selective bias. The WBCc as a common inflammatory biomarker has an advantage predictive of short-term outcome, whereas the association between baseline WBCc and long-term survival rate seemed limited.

## Conclusion

The WBCc is such a widely available inflammatory biomarker with the features of being stable, standardized, economical, and fast. The elevated WBCc represented emergency and extensive involvement of the aortic dissection. The high mortality rate was observed in patients presenting with the leukocytosis in circumstance of circulatory arrest. The preoperative WBCc more than 11.5 × 10^9^/L could be treated as potential predictor for surgical outcome of TAAD with adequate sensitivity and specificity.

## Data Availability

The datasets used and/or analyzed during the current study are available from the corresponding author on reasonable request.

## Abbreviations

CABG: Coronary artery bypass graft
CPB: Cardiopulmonary bypass
PaO2/FiO2: Arterial partial pressure of oxygen/fraction of inspired oxygen ratio
PND: Permanent neurologic dysfunction
ROC: Receiver operating characteristic curves
TAAD: Acute Stanford type A aortic dissection
WBCc: The white blood cell count

## References

[1] Conzelmann LO, Weigang E, Mehlhorn U, Abugameh A, Hoffmann I, Blettner M (2016). Mortality in patients with acute aortic dissection type a: analysis of pre- and intraoperative risk factors from the German registry for acute aortic dissection type a (GERAADA). Eur J Cardiothorac Surg.

[2] Bayegan K, Domanovits H, Schillinger M, Ehrlich M, Sodeck G, Laggner AN (2001). Acute type a aortic dissection: the prognostic impact of preoperative cardiac tamponade. Eur J Cardiothorac Surg.

[3] Ji Q, Lai H, Sun Y, Luo Z, Liu L, Liu C (2017). Impact of Presurgical mild acute respiratory distress syndrome on surgical mortality after surgical repair of acute type a aortic dissection. Int Heart J.

[4] Lingzhi C, Hao Z, Weijian H, Gaoshu Z, Chengchao S, Changxi C (2016). Outcome predictors in patients presenting with acute aortic dissection. J Cardiothorac Vasc Anesth.

[5] Asadollahi K, Hastings IM, Beeching NJ, Gill GV, Asadollahi P (2011). Leukocytosis as an alarming sign for mortality in patients hospitalized in general wards. Iran J Med Sci.

[6] Beasley TM, Schumacker RE (1995). Multiple regression approach to analyzing contingency tables: post hoc and planned comparison procedures. J Exp Educ.

[7] Trimarchi S, Nienaber CA, Rampoldi V, Myrmel T, Suzuki T, Mehta RH (2005). Contemporary results of surgery in acute type a aortic dissection: the international registry of acute aortic dissection experience. J Thorac Cardiovasc Surg.

[8] Pompilio G, Spirito R, Alamanni F, Agrifoglio M, Polvani G, Porqueddu M (2001). Determinants of early and late outcome after surgery for type a aortic dissection. World J Surg.

[9] Pansini S, Gagliardotto PV, Pompei E, Parisi F, Bardi G, Castenetto E (1998). Early and late risk factors in surgical treatment of acute type a aortic dissection. Ann Thorac Surg.

[10] Goda M, Imoto K, Suzuki S, Uchida K, Yanagi H, Yasuda S (2010). Risk analysis for hospital mortality in patients with acute type a aortic dissection. Ann Thorac Surg.

[11] Concistre G, Casali G, Santaniello E, Montalto A, Fiorani B, Dell'Aquila A (2012). Reoperation after surgical correction of acute type a aortic dissection: risk factor analysis. Ann Thorac Surg.

[12] Sbarouni E, Georgiadou P, Analitis A, Voudris V (2015). High neutrophil to lymphocyte ratio in type a acute aortic dissection facilitates diagnosis and predicts worse outcome. Expert Rev Mol Diagn.

[13] He R, Guo DC, Estrera AL, Safi HJ, Huynh TT, Yin Z (2006). Characterization of the inflammatory and apoptotic cells in the aortas of patients with ascending thoracic aortic aneurysms and dissections. J Thorac Cardiovasc Surg.

[14] Koren-Morag N, Tanne D, Goldbourt U (2005). White blood cell count and the incidence of ischemic stroke in coronary heart disease patients. Am J Med.

[15] Tshomba Y, Coppi G, Marone EM, Bertoglio L, Kahlberg A, Carlucci M (2012). Diagnostic laparoscopy for early detection of acute mesenteric ischaemia in patients with aortic dissection. Eur J Vasc Endovasc Surg.

[16] Takahashi T, Hasegawa T, Hirata N, Endo A, Yamasaki Y, Ashida K (2014). Impact of acute kidney injury on in-hospital outcomes in patients with DeBakey type III acute aortic dissection. Am J Cardiol.

[17] Guan X, Gong M, Wang X, Zhu J, Liu Y, Sun L (2018). Low preoperative fibrinogen level is risk factor for neurological complications in acute aortic dissection. Medicine (Baltimore).

[18] Shenaq SA, Yawn DH, Saleem A, Joswiak R, Crawford ES (1986). Effect of profound hypothermia on leukocytes and platelets. Ann Clin Lab Sci.

[19] Engels M, Bilgic E, Pinto A, Vasquez E, Wollschlager L, Steinbrenner H (2014). A cardiopulmonary bypass with deep hypothermic circulatory arrest rat model for the investigation of the systemic inflammation response and induced organ damage. J Inflamm (Lond).

[20] Duan XZ, Xu ZY, Lu FL, Han L, Tang YF, Tang H (2018). Inflammation is related to preoperative hypoxemia in patients with acute Stanford type a aortic dissection. J Thorac Dis.

[21] Chen MF, Chen LW, Cao H, Lin Y (2016). Analysis of risk factors for and the prognosis of postoperative acute respiratory distress syndrome in patients with Stanford type a aortic dissection. J Thorac Dis.

